# Trends in the characteristics of acute primary angle closure in Korea over the past 10-years

**DOI:** 10.1371/journal.pone.0223527

**Published:** 2019-10-09

**Authors:** Jun Young Ha, Mi Sun Sung, Hwan Heo, Sang Woo Park

**Affiliations:** 1 Department of Ophthalmology and Research Institute of Medical Sciences, Chonnam National University Medical School and Hospital, Gwangju, South Korea; 2 Center for Creative Biomedical Scientists, Chonnam National University, Gwangju, South Korea; Universidad de Monterrey Division de Ciencias de la Salud, MEXICO

## Abstract

**Purpose:**

To investigate the changes in the demographic, clinical, and biometric characteristics of APAC patients in South Korea during the last decade.

**Methods:**

Medical records of patients with APAC who visit the emergency department or the glaucoma clinic of Chonnam National University Hospital, a tertiary referral center in Gwangju, South Korea in 2007 and 2017 were analyzed. Demographics, clinical characteristics, and treatment modality were compared between the APAC patients in 2007 and 2017.

**Results:**

The number of patients with APAC increased from 54 in 2007 to 68 in 2017. Female patients in their 60s were most common in both groups and there was no significant difference in IOP, cataract grade, gonioscopic grading, PAS, or optic nerve damage between the two groups at baseline visit (all *P* > 0.05). However, APAC eyes in 2017 had a shallower ACD (1.74 ± 0.28 mm vs 1.87 ± 0.35 mm; *P* = 0.024) and greater LV (1.05 ± 0.26 mm vs 0.93 ± 0.19 mm; *P* = 0.001) than those of APAC eyes in 2007. During one year follow-up, 25 patients (51.02%) received LPI only, and 18 patients (36.73%) required LE, and 6 patients (12.24%) required phacotrabeculectomy or sequential LE and trabeculectomy. However, in 2017, LPI alone was sufficient in 23 patients (38.33%), 29 patients (48.33%) required further LE, and 8 patients (13.33%) required phacotrabeculectomy or sequential LE and trabeculectomy for the treatment of APAC (*P* = 0.015).

**Conclusions:**

Compared to older cases of APAC, recent cases received LE more frequently, which suggests an increasing trend of LE as a treatment option for APAC. In addition, recent cases had a greater LV and shallower ACD than older cases and these biometric differences may be one of the reasons for increasing rate of LE in this study.

## Introduction

Primary angle closure disease (PACD) is one of the leading causes of blindness worldwide and predominantly affects Asian populations [1,2]. Eyes with PACD are characterized by a crowded anterior segment and appositional or synechial closure of the angle. Acute primary angle closure (APAC) is a subtype of PACD and an ocular emergency that requires immediate reduction of the elevated intraocular pressure (IOP) to prevent permanent vision loss. In addition, APAC usually presents as a sequential disease, frequently involving the fellow eye [3].

In recent years, our understanding of the pathogenesis of APAC has expanded rapidly. Owing to anterior segment optical coherence tomography (AS-OCT), high-resolution cross-sectional images of the anterior segment can be obtained. These objective and reproducible techniques enable APAC to be sub-classified based on the principal mechanism of the disease. Ng et al [4] suggested that APAC can be classified into four different mechanisms, including pupillary block (PB), plateau iris syndrome, lens disproportion, and ciliary block.

Management of APAC has been personalized based on its principal mechanism. Traditionally, PB is considered to be main mechanism of APAC; therefore, laser peripheral iridotomy (LPI), which can break the PB, has been the standard treatment for APAC [5]. Despite a patent LPI, approximately 20% of APAC eyes suffered from residual angle closure and increased IOP in long-term follow-up, and required additional procedures [6,7]. Therefore, other factors such as non-PB mechanisms have been suggested to play an important role in some APAC subtypes. Among the non-PB mechanisms, exaggerated lens vault (LV) has been reported as an important anatomical risk factor for APAC. Moghimi et al [8,9] reported exaggerated LV as the responsible mechanism in approximately half of all APAC eyes.

Lens extraction (LE) with intraocular lens (IOL) implantation has progressively gained popularity in the treatment of APAC. It has been reported that LE relieves crowding of the AC by the lens, removes the PB, and prevents the formation of peripheral anterior synechia (PAS) in APAC [10,11]. In addition, multiple prospective studies have shown that LE is more effective in IOP control than LPI in APAC eyes with and without cataract [12–14].

Diagnosis and treatment of APAC have been evolving for long time. Although many studies have been conducted on the biometric features of APAC eyes and the efficacy of numerous therapeutic options, few studies have focused on the changing trend in the characteristics and management of APAC patients [13–16]. Therefore, this study aims to compare the demographic, clinical, and biometric characteristics of APAC patients who visited a tertiary referral center in South Korea in 2007 and 2017.

## Materials and methods

This retrospective study was conducted in accordance with the tenets of the Declaration of Helsinki and was approved by the Institutional Review Board (IRB) of Chonnam National University Hospital (CNUH) (IRB number: CNUH-2018-256). Medical records of patients with APAC who visit the emergency department or the glaucoma clinic of CNUH in Gwangju, South Korea in 2007 and in 2017 were analyzed. Demographics, clinical characteristics, and treatment modality were compared between the APAC patients in 2007 and 2017. The requirement to obtain written informed consent was waived by the IRB of CNUH, because our study was retrospective research based on medical records, and also because this research presented no more than minimal risk of harm to participants.

### Inclusion and exclusion criteria

Patients with APAC had to satisfy all four of the following criteria to be included in the study. (a) at least two of the symptoms of an acute IOP elevation: ocular or peri-ocular pain, nausea and/or vomiting, halos; (b) clinical signs: conjunctival injection, microcytic corneal edema, mid-dilated pupil, and shallow AC; (c) IOP at presentation of at least 30 mmHg; (d) presence of occludable angle, confirmed by gonioscopy [17]. Occludable angle closure was defined if the posterior trabecular meshwork (TM) could not be visualized in at least 3 quadrants.

Eyes with iris or angle neovascularization, pseudoexfoliation, lens intumescence or subluxation, or any iris or corneal abnormalities were excluded.

### Examinations

All patients underwent a complete ophthalmic examination including a medical and ocular history, measurement of best-corrected Snellen visual acuity, auto-refraction with keratometer (KR8800; Topcon, Tokyo, Japan), slit-lamp examination of anterior segment and posterior pole (optic disc and macula), IOP measurement with Goldmann applanation tonometry, stereoscopic optic disc photography, retinal nerve fiber layer photography, and Lenstar optical biometer (Haag-Streit AG, Koeniz, Switzerland) for measurement of axial length (AXL), and lens thickness (LT). Static and dynamic gonioscopy was performed in a dark room by a glaucoma specialist (S.W.P) using a Posner 4-mirror gonioprism (Ocular Instruments, Bellevue, WA, USA). Gonioscopic grading of the angle was done according to the following system: 0, none of the angle structures were visible; 1, only Schwalbe’s line and non-pigmented anterior TM were visible; 2, posterior TM could also be seen; 3, scleral spur (SS) could be detected; and 4, all angle components including Schwalbe’s line, TM, SS, and ciliary band were visible [18].

### Anterior segment optical coherence tomography imaging

All patients were imaged using AS-OCT (Visante OCT, ver. 2.0; Carl Zeiss Meditec, Dublin, CA, USA) under the same dark conditions by a single experienced operator. No treatments were started prior to imaging. The scans were centered on the pupil and horizontal cross-sectional images of the nasal and temporal angle (0–180°) were obtained until the quality was deemed sufficient for analysis. A single examiner (S.W.P) selected the best images with no motion artifacts, good visibility of the SS, and no image artifacts from the eyelids. Another glaucoma specialist examiner (J.Y.H) who were blinded to other clinical information, analyzed the images using custom software (Iridocorneal module, Carl Zeiss Meditec) [19,20]. Images with poor quality or inability to locate scleral spurs were excluded.

**Fig 1:**
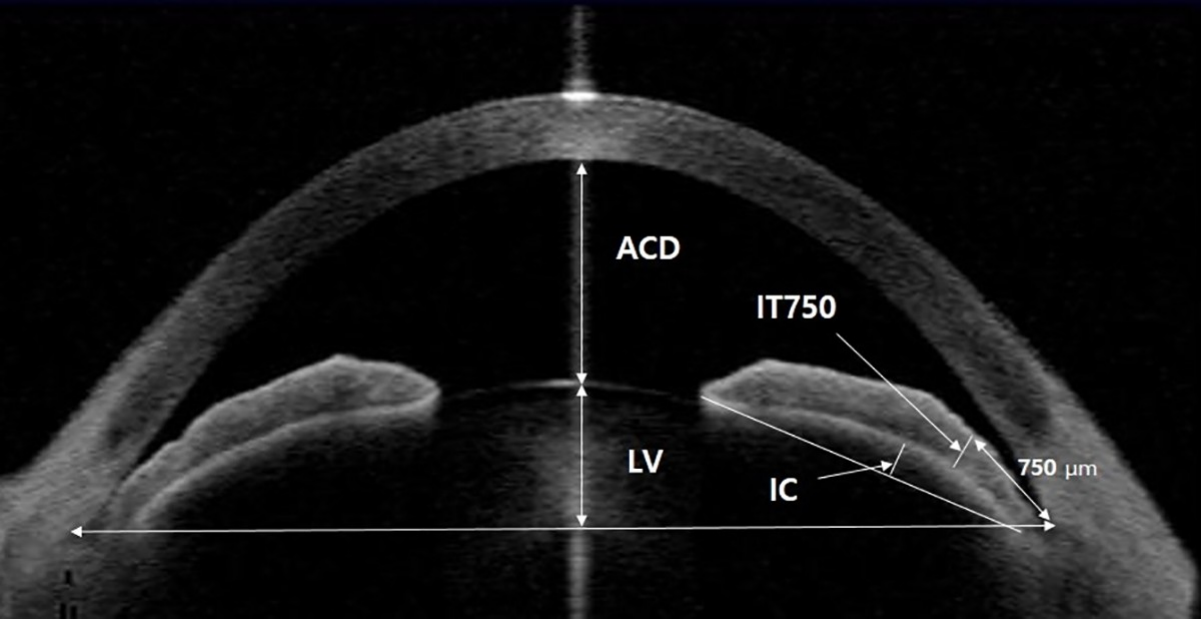
AS-OCT image showing the measurement of ACD, LV, IC, and IT750. ACD = anterior chamber depth; LV = lens vault; IC = iris curvature; IT750 = iris thickness at 750 μm from the SS.

We measured four parameters including anterior chamber depth (ACD), defined as the distance from the corneal endothelium to the anterior lens surface, LV, defined as the maximum perpendicular distance between the anterior pole of the crystalline lens and the horizontal line connecting the two SSs [21], iris curvature (IC), defined as the maximum perpendicular distance between the iris pigment epithelium and the line connecting the most peripheral to the most central point of the epithelium [22], and iris thickness at 750 μm from the SS (IT750) [23] (Fig 1).

### Treatment protocol

All patients were managed by a single protocol initially. Detailed laser and surgical procedures are described in Table 1. First, we administered medical treatment for IOP control, including topical brimonidine and fixed combination of timolol/dorzolamide, and systemic hyperosmotic agents (intravenous mannitol, 1 mg/kg). Laser peripheral iridotomy was performed for all patients as soon as the cornea permitted good visualization. Lens extraction was as performed in case of uncontrolled IOP (IOP > 25mmHg) and significant residual appositional angle closure (> 180 degree) despite the patient PI and using > 3 topical eyedrops. Trabeculectomy was performed in cases of uncontrolled IOP due to significant PAS (> 270 degree) or progression of glaucomatous optic nerve damage after LPI and LE. When performing trabeculectomy, combined phacotrabeculectomy was performed only in patients with visually significant cataract (presence of nucleus sclerosis, cortical cataract, or subcapsular cataract; visual acuity < 20/50; and affecting activities of daily living). Lens extraction was not considered in patients with clear lens during trabeculectomy.

**Table 1:** Interventions for management of acute primary angle closure in this study.

Intervention	Procedure
Laser peripheral iridotomy	• Laser peripheral iridotomy was performed in the superior region using sequential argon and Nd:YAG laser therapy after pretreatment with 2% pilocarpine instilled the eye. Argon settings of 500 to 1000 mW with a spot size of 50 μm for a duration of 0.05 s and a Nd:YAG setting of 2 to 5 mJ was used. • Topical steroid was prescribed and subsequently tapered slowly until the inflammation in the anterior eye segment had fully subsided.
Lens extraction	• Lens extraction was performed via phacoemulsification with IOL implantation. Phacoemulsification was performed under topical or retrobulbar anesthesia through a temporal 2.8-mm corneal incision with implantation of a foldable one-piece acrylic IOL (AcrySof, Alcon Laboratories Inc., Fort Worth, TX, USA) into the capsular bag. • Postoperatively, topical antibiotics and steroid were administered four times daily for 1 week and then tapered over 4 to 6 weeks. • Any glaucoma eyedrops were tapered if the mean IOPs at 2 consecutive visits were 21 mmHg.
Trabeculectomy & Phacotrabeculectomy	• All patients underwent fornix-based trabeculectomy with adjunctive mitomycin C. The scleral flap was rectangular, with a depth of dissection of approximately two-thirds of the scleral thickness. Cellulose sponge fragments soaked in mitomycin C (0.4 mL/mL) were placed between the dissected conjunctiva and the scleral flap for 3–5 min. After the excision of a block of corneoscleral tissue, peripheral iridectomy was performed and the rectangular scleral flap was closed with two 10–0 nylon stitches. Meticulous closure of the conjunctiva with 10–0 nylon stitches was performed to achieve a watertight wound. • In case of phacotrabeculectomy, similar steps for trabeculectomy were performed, but after irrigation of mitomycin C, temporal clear corneal phacoemulsification with intraocular lens implantation was performed. Sclerostomy and subsequent surgical steps were then performed as previously described.

Nd:YAG = neodymium–yttrium–aluminum–garnet; IOL = intraocular lens; IOP = intraocular pressure

### Statistical Analysis

Statistical analyses were performed using the Statistical Package for the Social Sciences, version 19 (SPSS Inc., Chicago, IL, USA). Continuous variables were expressed as the mean ± standard deviation and categorical data were presented as numbers (n) and percentages (%). Categorical variables were compared using the χ^2^ test and continuous variables were compared using the Student *t*-test. A *P*-value of less than 0.05 was considered statistically significant.

## Results

The demographic characteristics of patients with APAC in 2007 and 2017 are summarized in Table 2. Patients with APAC were increased from 54 in 2007 to 68 in 2017. In both groups, APAC patients were likely to be female and in their sixties. There was no significant difference in age and sex between the two groups (*P* = 0.868 and *P* = 0.450, respectively). APAC occurred at a similar frequency throughout all four seasons and no significant difference was observed between the two groups (*P* = 0.264).

**Table 2:** Demographic characteristics of patients with acute primary angle closure in 2007 and 2017.

	2007	2017	*P* value
Patients with APAC treated in CNUH	54	68	
Age (years)	63.83 ± 12.09	64.21 ± 12.32	0.868
40–49	4 (7.41)	6 (8.82)	
50–59	13 (24.07)	14 (20.59)	
60–69	20 (37.04)	25 (36.76)	
70–79	12 (22.22)	17 (25.00)	
≥ 80	5 (9.26)	6 (8.82)	
Sex			0.450
Male / Female	16 / 38 (29.63 / 70.37)	18 / 50 (36.00/64.00)	
Attack season			0.264
Spring (Mar, Apr, May)	12 (22.22)	18 (26.47)	
Summer (Jun, Jul, Aug)	14 (25.93)	17 (25.00)	
Fall (Sep, Oct, Nov)	13 (24.07)	15 (22.06)	
Winter (Dec, Jan, Feb)	15 (27.78)	18 (26.47)	

Values are presented as number (%) or mean ± standard deviation. CNUH = Chonnam National University Hospital

Baseline clinical characteristics of APAC patients in 2007 and 2017 were summarized in Table 3. There was no significant difference in IOP, cataract grade, gonioscopic grading, PAS, or optic nerve damage between the two groups (all *P* > 0.05).

**Table 3:** Clinical characteristics of patients with acute primary angle closure in 2007 and 2017.

	2007 (n = 54)	2017 (n = 68)	*P* value
Intraocular pressure (mmHg)	47.31 ± 9.23	44.53 ± 9.56	0.108
Lens nucleus opacity (per LOCS III)	2.54 ± 0.71	2.69± 0.73	0.265
Gonioscopy (inferior quadrant)	0.43 ± 0.49	0.46 ± 0.50	0.742
Grade A	31 (57.41)	37 (54.41)	
Grade B	23 (42.59)	31 (45.59)	
Grade C	0 (0)	0 (0)	
Grade D	0 (0)	0 (0)	
Grade E	0 (0)	0 (0)	
PAS (quadrants)	0.72 ± 1.03	0.59 ± 0.93	0.543
Vertical C/D ratio	0.42 ± 0.19	0.38 ± 0.18	0.269

Values are presented as number (%) or mean ± standard deviation. PAS = peripheral anterior synechiae; C/D, cup-to-disc; LOCS III, lens opacities classification system III. Gonioscopy A = open to Schwalbe’s line; B = open anterior to trabecular meshwork; C = open to posterior trabecular meshwork; D = open to scleral spur; E = open to ciliary body band

The measurments of biometric parameters of APAC are presented in Table 4. Notably, APAC eyes in 2017 had a shallower ACD (1.74 ± 0.28 mm vs 1.87 ± 0.35 mm; *P* = 0.024) and greater LV (1.05 ± 0.26 mm vs 0.93 ± 0.19 mm; *P* = 0.005) than those of APAC eyes in 2007. To investigate the distribution of APAC eyes according AXL in this study, APAC eyes in each group were categorized as short AXL (<22.5 mm), intermediate AXL (≥22.5 to <23.5mm), and long AXL (≥23.5mm) [24]. The median of AXL of APAC in 2007 and in 2017 were 22.40 mm and 22.42 mm, respectively (P = 0.865). There was no difference in distribution of APAC eyes according to AXL between the two groups (P = 0.799). There was no difference in other parameters between the two groups.

**Table 4:** Biometric parameters of acute primary angle closure in 2007 and 2017.

	2007 (n = 54)	2017 (n = 68)	*P* value
Km (D)	44.03 ± 1.17	44.34 ± 1.13	0.199
AXL (mm)	22.38 ± 1.15	22.46 ± 1.13	0.701
AXL (mm)	22.40 (21.60, 23.30)	22.42 (22.00, 23.29)	0.865
Range of AXL (mm)			0.799
< 22.5 mm	29 (54.17)	34 (50.00%)	
22.5 ≥ to <23.5 mm	16 (29.17)	24 (35.29%)	
≥ 23.5 mm	9 (16.67)	10 (14.71%)	
ACD (mm)	1.87 ± 0.35	1.74 ± 0.28	**0.024**
ACD (mm)	1.84 (1.62, 2.03)	1.67 (1.55, 1.91)	**0.032**
LT (mm)	4.68 ± 0.65	4.88 ± 0.63	0.088
LV (mm)	0.93 ± 0.19	1.05 ± 0.26	**0.005**
LV (mm)	0.88 (0.81, 1.02)	0.99 (0.87, 1.18)	**0.008**
IC (mm)	0.21 ± 0.06	0.19 ± 0.06	0.070
IT750 (mm)	0.44 ± 0.08	0.42 ± 0.09	0.203

Values are presented as number (%), mean ± standard deviation, or median (interquartile range). P values < 0.05 are bold. Km = mean keratometry; AXL = axial length; ACD = anterior chamber depth; LT = lens thickness; LV = lens vault; IC = iris curvature; IT750 = iris thickness at 750 μm from scleral spur.

**Fig 2:**
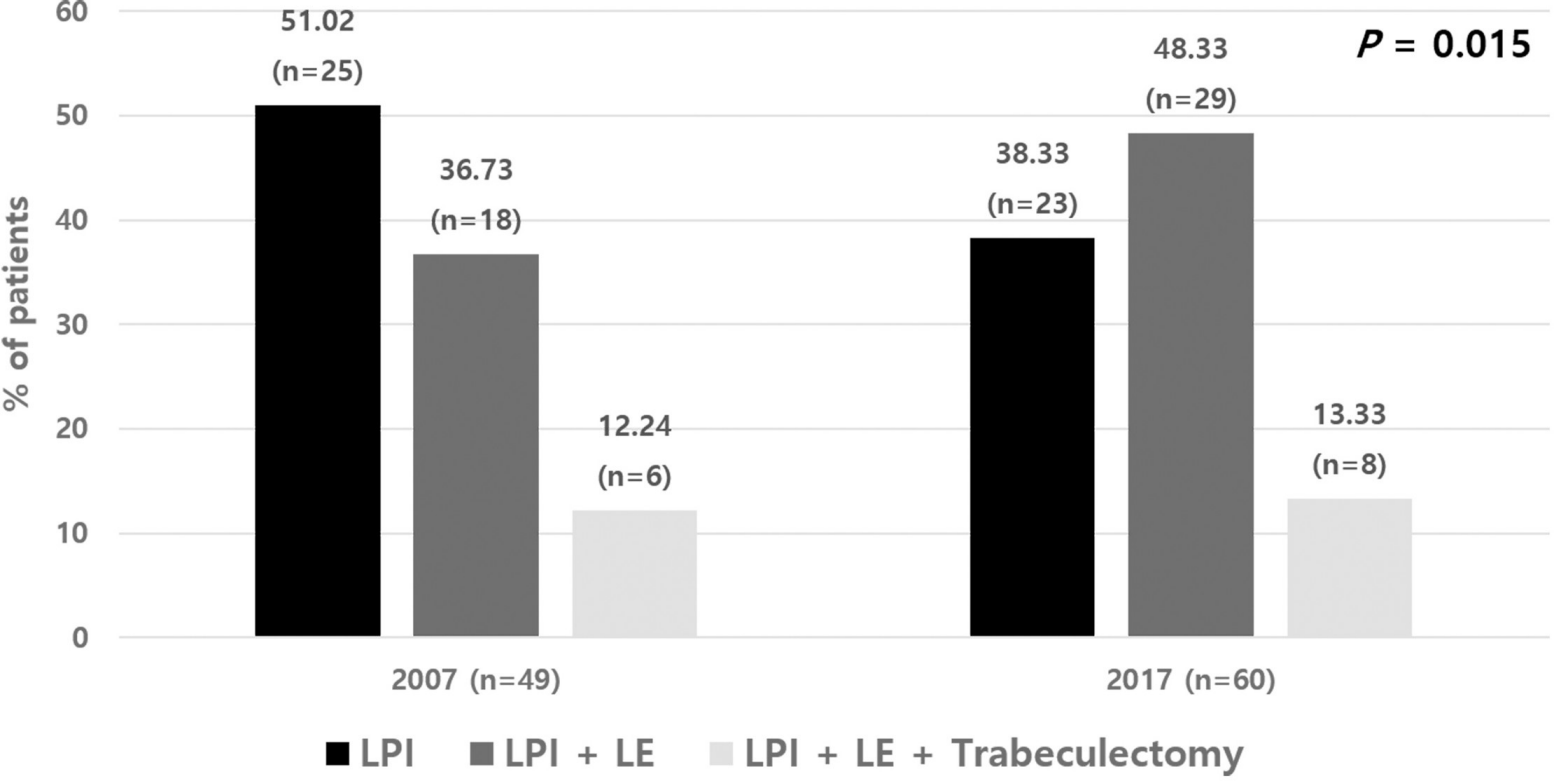
Comparison of treatment modalities between patients with APAC in 2007 and 2017. In 2007, of 49 patients, 25 patients (51.02%) underwent LPI only, 18 patients (36.73%) required further LE, and 6 patients (12.24%) required further trabeculectomy. However, in 2017, of 60 patients, only 23 patients (38.33%) underwent LPI, with 29 (48.33%) and 8 (13.33%) patients received LE and trabeculectomy, respectively, for the treatment of APAC (*P* = 0.015). APAC = acute primary angle closure; LE = lens extraction; LPI = laser peripheral iridotomy.

Treatment modalities of APAC were compared between the two groups (Fig 2). Five patients in 2007 and 8 patients in 2017 were excluded due to less than one year follow-up. During one year, 25 patients (51.02%) received LPI only, and 18 patients (36.73%) required LE, and 6 patients (12.24%) required phacotrabeculectomy or sequential LE and trabeculectomy. However, in 2017, LPI alone was sufficient in 23 patients (38.33%), 29 patients (48.33%) required further LE, and 8 patients (13.33%) required phacotrabeculectomy or sequential LE and trabeculectomy for the treatment of APAC (*P* = 0.015).

## Discussion

To the best of our knowledge, this is the first study to investigate the changes in demographical, clinical, and biometric characteristics of APAC in Korean patients. In this study, APAC patients were most common in female and in their sixties. Compared to older cases of APAC, recent cases received LE more frequently, which suggests an increasing trend of LE as a treatment option for APAC. In addition, recent cases had a greater LV and shallower ACD than older cases and these biometric differences may be one of the reasons for increasing rate of LE in this study.

Although there was no difference in IOP, cataract grade, LT, or gonioscopic evaluation between the two groups, recent cases showed a shallower ACD and greater LV than older cases in AS-OCT imaging. Especially, LV was more prominent in recent cases than older cases (*P* = 0.001). Traditionally, short AXL, shallow ACD, small corneal diameter, and a thicker and more anteriorly positioned lens have been considered as risk factors for APAC. However, several studies on Chinese, Japanese, and Iranian patients with angle closure reported that only LV was significantly associated with angle closure after multivariate adjustments for other clinical and lens factors such as age, sex, LT, lens position (ACD + 1/2LT), and relative lens position (lens position/AXL) [25–27]. Lens vault is one of AS-OCT parameters that measure the amount of lens that is located anterior to the plane of the scleral spurs. In other words, LV is true portion of lens in anterior chamber. Increased thickness of the lens anterior to the plane of the scleral spurs pushes the peripheral iris against the TM, directly worsening angle crowding or induces PB by expanding iridolenticular contact and narrowing the iris–lens channel [26]. Consistent with these results, our data also show that importance of LV than other biometric parameters in pathogenesis of APAC.

These biometric difference may be due to differences in the distribution of subclasses of APAC between the two groups. The pathogenesis of PACD has been studied extensively, and PACD is now considered as a group of diseases consisted of different mechanisms of resistance to aqueous outflow. Recently, many studies have classified PACD into three characteristic subgroups using a hierarchical clustering method, namely, predominant LV component, predominant iris component, or a mixture of both [13,17,28]. The predominant LV component subgroup is characterized by greater LV, shallower ACD, and normal iris parameters. However, the predominant iris component subgroup has a large iris area or thicker iris but had a relatively small LV with deeper ACD. In this study, APAC patients in 2017 might be more comprised of “predominant LV subgroup” than those in 2007. Both groups showed an increased LV (1.05 ± 0.26 mm in 2017 and 0.93 ± 0.19 mm in 2007), which are similar level with “predominant LV subgroup” of APAC in other studies (i.e., 1.11 ± 0.24 mm in Moghimi et al [17], and 1.10 ± 0.29 mm in Baek et al [13]), but APAC eyes in 2017 had a greater LV than those in 2007. In addition, both groups had a shallow ACD (1.74 ± 0.28 mm in 2017 and 1.87 ± 0.35 mm in 2007), which are also concurrent with “predominant LV subgroup” of APAC in other studies (i.e., 1.72 ± 0.12 mm in Moghmi et al [17], and 1.82 ± 0.38 mm in Baek et al [13]), but APAC eyes in 2017 showed an even shallower ACD that those of 2007. Moreover, there was no significant difference in IC or IT750 between the two groups. Based on these findings, increased LV might be the main pathogenesis of both groups in this study but APAC in 2017 might have more proportion of increased LV subgroup than those in 2007.

Considering these biometric differences in APAC eyes in 2007 and 2017, the difference in treatment modalities is not surprising. The proportion of APAC patients who can be treated with medication and LPI decreased from 51.02% in 2007 to 38.33% in 2017, and who further required LE increased from 36.73% in 2007 to 48.33% in 2017 in this study. Although LPI has been considered as standard treatment and can considerably increase the central ACD, and AC area by eliminating PB component in APAC eyes [15], several studies reported that LPI may not always be enough for the long-term treatment of APAC and a high portion of LPI-treated patients eventually develop PAS and/or elevated IOP despite patent PI. This suggests the importance of non-PB mechanisms in pathogenesis of APAC [29–32]. Suwan Y et al [29] highlighted the presence of a substantial proportion of non-PB mechanisms underlying APAC in Thai population and most common mechanism was crowded angle by iridolenticular wrapping, which was similar feature to an increased LV in this study, such as shallow both central and peripheral ACD, and increased iridolenticular contact distance. By directly addressing the lens, LE can manage both PB and non-PB mechanisms. Many prospective, longitudinal studies have reported the superior effectiveness of LE over LPI in the improvement of anterior chamber angle parameters and control of IOP in APAC eyes with cataracts [14,16,31,32]. Moreover, recent EAGLE trial reported that even clear LE showed greater efficacy, safety, and cost-effectiveness than LPI with topical medication as a first-line treatment in patients over the age of 50 with early PACD with high IOP or PACG [12]. Because our data showed the results of only one year follow-up, the need for LE is expected to grow in the future.

This study should be interpreted with its limitations in mind. First, this was a retrospective hospital-based study with a relatively small number of cases, and therefore may not be representative of the general APAC population or of other ethnic backgrounds. In addition, there might be surgical decision protocol confounder in this study. Recently, glaucoma surgeons are much more comfortable for LE as a treatment option for APAC, especially after EAGLE trial. Since this is not a prospective study and the treatment options are not randomly selected, the surgeon may be influenced by EAGLE trial [12]. However, the APAC eyes in this study were diagnosed and treated consistently with a single standard protocol by experienced glaucoma specialists throughout the study period. Therefore, we believe our results are appropriate for evaluating the changes during the study period. Second, only horizontal meridian images obtained using AS-OCT were used for analysis in this study; however, angle structure may not have been uniform over the entire 360 degrees and meridian-specific differences may have occurred. Nevertheless, superior and inferior angle images cannot be acquired without manipulation of the eyelid, which may result in compression of the angle structures. Third, measurements of AS-OCT parameters are obtained manually, which are prone to observer bias and errors. In fact, the identification of scleral spur (SS) is most important in measuring AS-OCT parameters, which are greatly influenced by the quality of the image. To acquire the best quality of image, all scans were performed by a single-well trained operator in a controlled environment. In addition, one glaucoma specialist (S.W.P) selected the best quality image with good visibility of SS and images with poor quality or inability to locate scleral spurs were excluded in this study. To minimize inter-observer variability, one experienced glaucoma specialist (J.Y.H) analyzed the selected images. Finally, the treatment results were not compared between the APAC eyes in the two groups. For the development of personalized care of patients with APAC, additional prospective longitudinal studies are required to compare the efficacy of treatment modalities based on the principal mechanism of APAC.

In conclusion, compared to older cases of APAC, recent cases received LE more frequently, which suggests an increasing trend of LE as a treatment option for APAC. In addition, recent cases had a greater LV and shallower ACD than older cases and these biometric differences may be one of the reasons for increasing rate of LE in this study.

## Supporting information

S1 Dataset Data of demographics, clinical characteristics, and biometric parameters of acute primary angle closure in 2007 and 2017. (XLSX)
